# Knowledge of Chosen Family History and Depressive Symptoms in Sexual Minority Women

**DOI:** 10.3389/fpsyg.2021.624641

**Published:** 2021-06-15

**Authors:** Jamie M. Gardella, McKenna F. Parnes, William Hirst, Adam D. Brown

**Affiliations:** ^1^Department of Psychology, The New School for Social Research, New York, NY, United States; ^2^Department of Psychology, Suffolk University, Boston, MA, United States; ^3^Department of Psychiatry, New York University School of Medicine, New York, NY, United States

**Keywords:** sexual minority women, sexual orientation, well-being, autobiographical memory, intergenerational memory

## Abstract

Recent work on intergenerational memory has revealed a positive association between family of origin knowledge and wellbeing in adolescents. However, little is known about the generalizability of these data, as significantly less attention has focused on autobiographical memory sharing and wellbeing in historically marginalized communities. Given the high incidence of familial rejection and abandonment within the LGBTQIA + community, close relationships with individuals outside of one’s family of origin, *chosen families*, often serve as an important source of social support. This study sought to examine the relationship between knowledge of a close non-family member and wellbeing among emerging adult sexual minority women (SMW) according to their gender presentation. A community sample from New York City comprised of heterosexual women (*n* = 50), masculine-presenting SMW (*n* = 50), and feminine presenting SMW (*n* = 50) completed measures associated with their knowledge of their family of origin, knowledge of a close non-family member, as well as self-reported measures of depression, emotion regulation, and socio-demographic questions. Family of origin knowledge was associated with lower levels of depression only among heterosexual women. However, heterosexual and SMW who knew more about their close non-family member reported lower levels of depression. Additionally, emotion regulation (cognitive reappraisals) mediated the relationship between knowing more about one’s chosen family and lower depressive symptom severity among heterosexual women, but this relationship was only significant for SMW who were at least moderately open about their sexuality. These findings extend the literature on the benefits of memory sharing to historically marginalized communities by showing that memory sources outside of one’s family of origin may be particularly important. Additionally, these data begin to shed light on potential mediating factors, such as emotion regulation and openness about one’s sexual identity, that underlie the links between memory sharing and metrics of wellbeing. Taken together, in contexts in which there may not be opportunities to learn about family history from one’s family of origin, it appears that access to stories from someone close outside of one’s family is also associated with lower levels of depression.

## Introduction

Considerable research has indicated that autobiographical memories play a variety of roles in supporting wellbeing, and alternations in both the characteristics and content of such memories is associated with a wide range of mental health disorders (Moore and Zoellner, 2007; Williams et al., 2007; Morgan, 2010; Sumner, 2012; Zhang et al., 2019). Autobiographical memories may contribute to positive mental health because personal narratives support functions such as decision-making, establishing and maintaining social support, and provide a sense of self-continuity (Bluck et al., 2005; Bluck and Alea, 2011; Vranić et al., 2018).

Within the field of autobiographical memory research, greater emphasis is now being paid to the social dynamics of memory. Although individuals may retrieve personal memories alone, autobiographical memories are often recalled in the presence of others (Hirst and Echterhoff, 2012). There now exists considerable work demonstrating how autobiographical memories are shaped by a myriad of social dynamics and contexts (Hirst and Echterhoff, 2012; Fivush, 2018; Vlasceanu et al., 2018). Additionally, how memories are shared socially have been found to play key roles in a wide range of processes that bear on mental health, including, but not limited to, the onset and development of autobiographical memory (Nelson and Fivush, 2004), the construction of the self and gender identity (Reese et al., 1996; Pasupathi et al., 2007), decision-making (Coman et al., 2013), meaning making (McLean et al., 2007), and emotion regulation (Maswood et al., 2019).

One area of research within the study of socially-shared autobiographical memory that has been more directly linked to mental health outcomes has emerged from the study of intergenerational autobiographical memory transmission. Whereas it has long been identified that storytelling is a critical part of family interactions and occurs often (Miller, 1994), a burgeoning body of work has begun to examine some of the potential emotional and mental health benefits that might be linked with knowledge of one’s intergenerational history (Fivush, 2018). For example, two studies conducted with adolescent girls revealed a significant correlation between knowing more about one’s family history on a self-report measure called the Do You Know Scale (DYK; Duke et al., 2008) and wellbeing (Duke et al., 2008; Fivush et al., 2010). The DYK scale includes a set of 20 Yes/No questions testing knowledge for family information that a child could not possibly have learned first-hand (Duke et al., 2003). Therefore, higher scores on the DYK suggest that these individuals are more aware of their family history through indirect sources of information, such as family storytelling. To date, the extant literature has shown that greater knowledge of family history, as measured by the DYK, is significantly correlated with internal locus of control, higher self-esteem, better family functioning, greater family cohesiveness, lower levels of anxiety, and lower incidence of externalizing behaviors (Duke et al., 2008; Fivush et al., 2010). A related study found that family stories emerged over dinner with parents and their pre-adolescent children and reported that children of more engaged mothers had fewer internalizing behaviors, such as anxiety and depression (Fivush et al., 2011).

Although the mechanisms are not yet fully understood, some of the observed mental health benefits associated with intergenerational autobiographical memories appear to be especially important for wellbeing among girls and women. For instance, Zaman and Fivush (2011) explored the relationship between the frequency with which adolescents took a parent’s perspective and wellbeing when retelling intergenerational narratives. The findings revealed gender differences, as only adolescent girls who retold their mothers’ intergenerational narratives from their perspective were reported by their mothers as having fewer problematic behaviors in general (Zaman and Fivush, 2011) and they may be more inclined to derive meaning from intergenerational narratives than boys in this particular stage of development (Merrill and Fivush, 2016). When a similar study was conducted with emerging adults, higher coherence in retelling maternal intergenerational narratives was correlated with better wellbeing among both genders; however, only female emerging adults also demonstrated this association with paternal intergenerational narratives (Merrill et al., 2019). These results imply that intergenerational narratives may benefit both genders in emerging adulthood with some individual differences. Fivush et al. (2008) have speculated that those family rituals and traditions that often promote the sharing of intergenerational narratives may cultivate connection and emotional intimacy, which may confer a sense of stability and ultimately self-esteem on an individual level.

Although these results offer potential important insights for supporting wellbeing in families, these studies, to our knowledge, have focused on intact heteronormative families and have not explored populations who may have more complex family dynamics driven by reactions to stigmatized identities. The absence of such data leaves open important questions as to whether the same patterns observed in prior research generalize to families in which access to family histories, for a variety of reasons, may be less accessible, less likely to be shared, or if this knowledge translates into better mental health for the subsequent generation.

One population in particular that might benefit from understanding whether and how socially-shared family knowledge might be associated with mental health outcomes are sexual minority women (SMW)^1^. SMW are more likely to experience mental health conditions, such as depression, anxiety, substance misuse, and suicidality, in comparison to heterosexual women (King et al., 2008; McCabe et al., 2009; Bostwick et al., 2010; Everett et al., 2016; Kerridge et al., 2017). While other sexual minority (SM) groups also face a higher risk of experiencing mental health disparities, we elected to focus on SMW for several reasons. As findings from the extant intergenerational memory literature have been fruitful for adolescent girls, we decided to direct our attention to women in an effort to extend previous findings with gender. Additionally, SMW are underrepresented in the SM literature, and, concurrently, few studies have explored the role of gender non-conformity in stress processes that influence mental health (Everett et al., 2019).

Theoretical models of mental health disparities among sexual minorities have argued that these individuals’ wellbeing suffers in part because of stigma, prejudice, and discrimination (Meyer, 2003; Hatzenbuehler, 2009). We relied on the psychological mediation framework of SM stress advanced by Hatzenbuehler (2009) in generating our hypotheses, given its steady foundation of empirical support. This model suggests that maladaptive emotional regulatory processes partially mediate the relationship between stigma-related stress (e.g., discrimination) and psychopathology in the SM community. For instance, an experience of discrimination could initiate a maladaptive cognitive process, such as rumination, which in turn raises the likelihood of experiencing symptoms of depression or anxiety. However, the psychological mediation framework notably does not integrate factors promoting wellbeing. We posit that SMW may not show the same associations between knowledge of family history and metrics associated with wellbeing (i.e., depression in the present study). That is, even if SMW are aware of their family history, general stigma-related stress from their structural position in society coupled with the experience of familial rejection (or fear of it, if concealing their sexual identity) may hinder the processes believe to underlie the results obtained in previous research (e.g., Duke et al., 2008; Fivush et al., 2010).

On the other hand, although SMW may not show some of the same patterns between intergenerational memory and metrics associated with wellbeing within their family of origin, it is possible that knowledge of people’s histories outside of the “traditional family” context may serve as an alternative source of autobiographical memories that might influence metrics associated with wellbeing. Given the historical social marginalization of the broader SM community that was partially driven by religious beliefs and moral judgments, it was not uncommon for families to reject their adult children on the basis of their sexual identities, and more importantly, for individuals to conceal their sexual identities to avoid rejection. Beginning in the 1970s, the concept of a “*chosen family*” emerged as a partial explanation for the resilience cultivated among SMs who have either a physical or intrapsychic separation from their families of origin (Dewaele et al., 2011; Blair and Pukall, 2015; Hull and Ortyl, 2019). A chosen family consists of non-biologically related close friends whose roles intersect with those traditionally held by members of one’s family of origin (Weston, 1991; Dewaele et al., 2011). Although support for SMs has increased among many societies and families over the past 50 years, the understudied chosen family remains a critical tool for SM mental health and wellbeing. Importantly, the concept of a chosen family has evolved to include families of origin in addition to friends and romantic partners (Nardi, 1999). To date, to our knowledge, no studies have investigated the relations between intergenerational autobiographical knowledge and depressive symptoms in relation to a member of one’s chosen family.

As a marker of wellbeing and mental health, autobiographical family knowledge is believed to reflect certain cognitive, emotional, and relational processes that exist in families in whom histories are freely shared (Duke et al., 2008). Such families operate as a cohesive unit, support each other, and demonstrate high levels of narrative co-construction. Considering that the psychological mediation framework does not account for protective factors in the SM stress process, we sought to extend it by evaluating two empirically demonstrated factors of wellbeing in SMs (i.e., degree of comfort with one’s sexual identity and quality of emotion regulation strategies) as mediating variables that have been identified as critical buffers against stress in this population. Although the original study by Duke et al. (2008) did not examine emotion regulation strategies as a mediator of the relationship between the processes underlying autobiographical family knowledge and wellbeing, there is ample evidence supporting emotion regulation, particularly cognitive reappraisal, as critical for psychiatric symptom reduction, irrespective of sexual orientation (Eftekhari et al., 2009). In accordance with the extant literature, we expect that the processes reflected by autobiographical family (of origin and chosen family) knowledge will only result in lower depressive symptoms in SMW who also exhibit comfort with their sexuality and who tend to deploy cognitive reappraisal emotion regulation strategies when faced with stress.

One way that one’s experience of sexual identity has been studied is through measures of *outness*, or the degree to which a SM is open about their sexual orientation. Although it is not a direct measure of SM discrimination, the degree to which a SMW is open about her sexual identity across contexts may reflect an overall sense of safety and comfort, which are important factors for coping with stress. In addition, while SMW who are more open about their sexual identity may feel a greater sense of comfort in authentic self-expression (Hutson, 2010; Clarke and Spence, 2013) they are also more visible and face a higher risk of encountering stigma-related stress, especially with a more masculine-leaning gender presentation (Skidmore et al., 2006; Rieger and Savin-Williams, 2012; Martin-Storey and August, 2016). As such, the association between outness, or the extent to which a SM is open about their sexual orientation with others, and mental health is nuanced (Feinstein et al., 2019). Some cross-sectional studies have reported a positive association between outness and mental health (e.g., Beals et al., 2009; Juster et al., 2013; Kosciw et al., 2015; Michaels et al., 2016), while others have reported higher rates of substance use with outness (Parks et al., 2007). This conflictual finding may be explained by the historical importance of bars in forging social connections in SM communities and its contribution to a normative perception of substance use (Hatzenbuehler et al., 2008a).

In addition, the quality of emotion regulation strategies has been implicated as an important correlate of several mental health conditions in adults, regardless of sexual orientation (Aldao et al., 2010; Kring and Sloan, 2010). With regard to SMs, a number of studies have shown that effective emotion regulation processes serve as a buffer against psychological distress resulting from discriminatory experiences (Feinstein et al., 2012; Brewster et al., 2013; Velez et al., 2013; Szymanski et al., 2014; Puckett et al., 2016; Kaufman et al., 2017). Recent work with SMs has implicated emotion regulation as a key mediator between minority stress and substance misuse in adults (Rogers et al., 2017) and between SM status and symptoms of depression and anxiety in adolescents (Hatzenbuehler et al., 2008b). Additionally, stress-related growth was linked to the use of more effective emotion regulation strategies and, thus, fewer internalizing symptoms (Wang et al., 2016).

### The Current Study

We sought to evaluate the positive association between the cognitive, emotional, and relational processes evidenced by intergenerational memory and metrics associated with wellbeing (i.e., level of depressive symptoms) that were previously reported by Duke et al. (2008) in emerging adult SMW. In order to account for the diversity of experiences reported by women with different gender presentations, we aimed to recruit both self-identified masculine-presenting (i.e., traditionally referred to as butch, or a woman who endorses having, or is recognized as having, behaviors, dress, style, or other aspects of an identity associated with masculinity by society; Rosario et al., 2008) and self-identified feminine-presenting (i.e., traditionally referred to as femme, or a woman who endorses having, or is recognized as having, behaviors, dress, style, or other aspects of an identity associated with femininity by society; Rosario et al., 2008) SMW. Given the history of familial rejection and continued importance of the chosen family in this population, we also hypothesized that chosen family knowledge would reflect similar underlying processes and correlate with lower levels of depressive symptoms in SMW who were more likely to use cognitive reappraisal emotion regulation strategies and who endorsed a higher degree of outness. We recruited heterosexual women for the purpose of comparison.

## Materials and Methods

### Participants

A total of 150 emerging adult women (i.e., between the ages of 18 and 29) were recruited *via* Craigslist, professional e-mail listservs, posted flyers in several targeted locations (e.g., The LGBT Community Center of New York City, lesbian bars, in various New School University buildings), respondent-driven sampling, and snowball sampling. Participants were remotely administered a questionnaire containing several measures and self-reported their age, gender, sexual identity, and gender presentation (SMW only). Based on their responses regarding their sexual identity (attracted to and have sex with women, men, or both) and/or gender presentation, they were stratified into one of the following groups: self-identified masculine-presenting SMW (*n* = 50), self-identified feminine-presenting SMW (*n* = 50), and heterosexual women (*n* = 50).

### Measures

#### “Do You Know?” Scale (Duke et al., 2008)

The DYK scale consists of 20 questions and was designed to measure family knowledge in adolescents. For example, *Do you know some of the lessons that your parents learned from good or bad experiences?* and *Do you know where some of your grandparents grew up?* Scoring consists of summing the total number of Yes responses, indicating knowledge of particular aspects of family history (Cronbach’s alpha = 0.80).

#### Chosen Family DYK Scale

The Chosen Family DYK scale was established to quantify historical knowledge about one’s closest friend, a proxy for chosen family. The scale is comprised of 21 questions, many of which are identical to original DYK items. As some items (e.g., *Do you know how your parents met?*) were not applicable, a Ph.D. clinical psychologist and several advanced graduate students in clinical psychology independently generated alternative items that would apply to a relationship with a close friend. The final two questions sought to measure the depth of the friendship (*Do you talk to this person about romantic relationships and sexual experiences?*) and congruence of sexual identity (*Does this person share the same sexual orientation as you?*). The 21-item scale was administered to a diverse group of students (both graduate and undergraduate) and was finalized following feedback. Scoring was identical to the original DYK scale (Cronbach’s alpha = 0.83).

#### Patient Health Questionnaire-9 (PHQ-9, Kroenke et al., 2001)

The Patient Health Questionnaire (PHQ) is a 9-item self-report measure used to screen for Major Depressive Disorder (Cronbach’s alpha = 0.88).

#### Emotion Regulation Questionnaire (Gross and John, 2003)

The Emotion Regulation Questionnaire (ERQ) is a 10-item scale designed to measure the tendency to regulate emotions through Cognitive Reappraisal and Expressive Suppression strategies. Participants are asked to rate each item on a 7-point Likert scale, ranging from 1 (strongly disagree) to 7 (strongly agree). Scoring results in the Cognitive Reappraisal facet (six items) (Cronbach’s alpha = 0.85) and the Expressive Suppression facet (four items) (Cronbach’s alpha = 0.75).

#### Outness Inventory (Mohr and Fassinger, 2000)

The Outness Inventory (OI) is an 11-item scale designed to measure the extent to which LGB individuals are open about their sexual orientation. Responses indicate the degree to which the participant’s sexual orientation is known by and openly discussed with various individuals, groups, and communities. Each item is rated on a scale ranging from 1 (person definitely does NOT know about your sexual orientation status) to 7 (person definitely knows about your sexual orientation status, and it is openly talked about). Respondents can also indicate if an item does not apply to them. Scoring produces an Overall Outness index (Cronbach’s alpha = 0.88).

### Statistical Analyses

**FIGURE 1:**
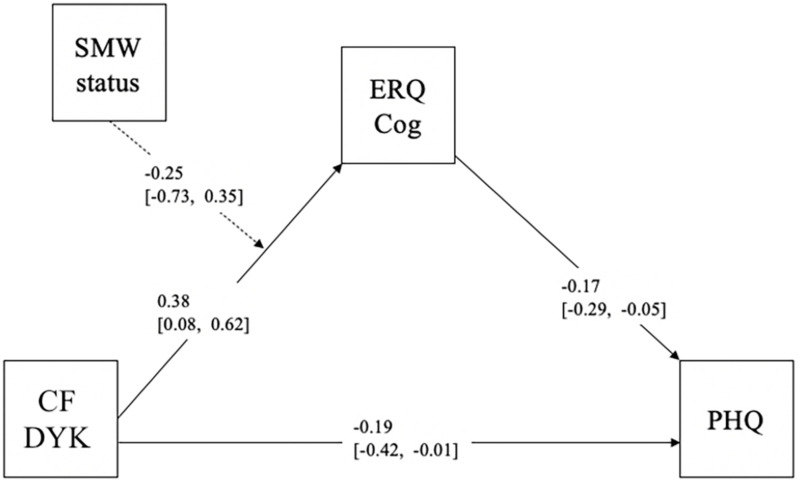
Overall model full sample. SMW, Sexual Minority Women Status; CF DYK, Chosen Family Do You Know; ERQ Cog, Emotion Regulation Questionnaire-Cognitive Reappraisals; PHQ, Patient Health Questionnaire-9.

Descriptive analyses were conducted in IBM SPSS statistics version 25 (IBM Corp, 1988–2017) and path analyses were carried out using Mplus version 8 (Muthén and Muthén, 1998–2017). Variables were assessed for skew, kurtosis, and normality. Considering the small sample size, path analysis with observed variables was conducted rather than creating latent variables for structural equation modeling (Webster-Stratton and Hammond, 1999; Alfaro et al., 2006; Bjørknes et al., 2012). Model fit was assessed based on chi-square test of model fit, root mean square error of approximation (RMSEA), comparative fit index (CFI), and the standardized root mean square residual (SRMR), following the recommended guidelines for each model fit index (see Hu and Bentler, 1999). Path analysis was used to examine the proposed relations among the predictor, mediator, moderator and outcome variables of interest. Figure 1 presents the conceptual model.

Main study hypotheses were tested using 95% bias-corrected bootstrap confidence intervals (CIs) estimated based on 1,000 random samples (Preacher and Hayes, 2008). This is the preferred method for mediation analysis because it controls the Type I error rate while maintaining high statistical power (MacKinnon et al., 2007). Significant indirect effects were probed by examining indirect effects at various levels of the moderators [i.e., outness overall at low (2.8), medium (4.66), and high (6.49) levels and sexual minority status (1) vs. heterosexual status (0)].

## Results

### Demographics and Preliminary Analyses

Of the 147 participants in total, no participants were missing data on variables included in the model. Three participants in the masculine-presenting SMW group were excluded from the dataset after not following instructions. Given that the data met the assumptions underlying the maximum likelihood (ML) method (e.g., multivariate normality), full information maximum likelihood estimation (FIML) was used to account for missing data in all models (Enders, 2011). Participants included 31.1% masculine-presenting SMW (*n* = 46), 34.5% feminine-presenting SMW (*n* = 51), and 34.5% heterosexual women (*n* = 50), with a mean age of (*M* = 24.58, SD = 3.01). Racial breakdown was as follows: 53.7% White/European, 12.9% Black/African American/Caribbean, 12.2% Hispanic/Latinx, 10.2% Asian/Pacific Islander, 9.5% Multiracial, and 1.4% Other. Over one-third of the sample (38.1%) completed a 4-year degree, while 29.3% completed some college, 12.2% have a professional degree, 10.9% have completed a 2-year degree, 5.4% have graduated high school, 2.7% have a doctorate degree, and 1.4% do not hold a high school or post-secondary degree.

#### Group Differences

One-way analyses of variance (ANOVAs) were conducted to evaluate between-group differences across demographic, clinical, emotion regulation, and DYK measures. No significant group differences were seen across groups for age, race, education level, ERQ scores, or PHQ scores (see Table 1).

**TABLE 1:** Comparison of means (standard deviations) of measures across groups.

**Measures**	**Masculine-SMW**	**Feminine-SMW**	**Hetero-women**
PHQ Scores	6.52 (5.80)	8.47 (5.80)	6.74 (5.06)
DYK Total	13.41 (3.99)	14.10 (3.87)	13.92 (3.55)
Chosen Family DYK	16.61 (4.14)	14.67 (4.30)	14.44 (4.15)
ERQ: Cognitive Reappraisal	31.50 (6.64)	31.16 (6.26)	29.56 (7.22)
ERQ: Emotional Suppression	15.33 (5.94)	14.36 (5.38)	13.96 (5.15)

*PHQ, Patient Health Questionnaire-9; DYK, Do You Know, and ERQ to Emotion Regulation Questionnaire.*

#### Original Do You Know

When looking at the original DYK scale, there were no significant between-group differences for DYK scores, and correlations were not seen between masculine-presenting SMW, feminine-presenting SMW, or heterosexual women and DYK scores (see Table 2). Consistent with previous research a significant correlation was found between level of depressive symptoms and DYK scores, but only among heterosexual women (*r* = −0.32, *p* = 0.02). Additionally, a significant correlation was seen between DYK scores and Outness (*r* = 0.21, *p* = 0.04) among SMW, such that individuals who knew more about their families of origin were more open about their sexual orientation.

**TABLE 2:** Zero-order correlations among study variables.

	**1**	**2**	**3**	**4**	**5**	**6**	**7**	**8**	**9**	**10**	**11**	**12**	**13**	**14**	**15**
1. Age	–														
2. Masculine-presenting women	0.11	–													
3. Feminine-presenting women	−0.49**	–0.09	–												
4. Hetero-women	−0.49**	−0.52**	–0.01	–											
5. Black/African American	0.00	0.15	–0.15	−0.17*	–										
6. White/European	0.07	–0.01	–0.05	−0.42**	0.12	–									
7. Asian/Pacific Islander	–0.08	–0.01	0.09	–0.13	−0.36**	0.00	–								
8. Hispanic/Latinx	0.02	–0.14	0.13	–0.14	−0.40**	–0.13	–0.07	–							
9. Multiracial	–0.02	0.01	0.01	–0.13	−0.35**	–0.11	–0.12	0.08	–						
10. Other	–0.08	0.04	0.04	–0.05	–0.13	–0.04	–0.04	–0.04	–0.05	–					
11. PHQ Scores	–0.09	0.16	–0.07	0.00	0.11	–0.09	–0.04	–0.05	0.02	–0.15	–				
12. DYK Total	–0.07	0.05	0.02	−0.17*	0.15	–0.12	0.00	0.06	–0.01	–0.05	0.22**	–			
13. Chosen Family DYK	0.22**	–0.09	–0.13	–0.11	0.03	–0.08	0.00	0.186*	–0.05	−0.19*	0.40**	0.04	–		
14. ERQ: Cognitive Reappraisal	0.08	0.05	–0.12	0.16	–0.06	–0.06	–0.01	0.00	–0.03	−0.23**	0.04	0.180*	0.09	–	
15. ERQ: Emotional Suppression	0.10	–0.02	–0.07	0.14	–0.14	0.14	0.02	–0.11	0.03	–0.06	–0.09	–0.09	0.00	–0.07	–
16. Outness	0.48**	0.52**	−0.99**	0.14	0.07	–0.10	–0.13	–0.02	–0.04	0.08	–0.01	0.12	0.09	0.09	0.03

PHQ, Patient Health Questionnaire-9; DYK, Do You Know; ERQ, Emotion Regulation Questionnaire. *P < 0.05; **P < 0.01.

#### Chosen Family Do You Know

There were two significant between-groups differences for Chosen Family DYK scores [*F*(2, 144) = 3.82, *p* = 0.024, η^2^ = 0.05]. Masculine-presenting SMW (*M* = 16.61, SD = 4.14) knew more about their chosen families compared to feminine-presenting SMW (*M* = 16.67, SD = 4.30) (Cohen’s *d* = 0.46) and heterosexual women (*M* = 14.44, SD = 4.14) (Cohen’s *d* = 0.52).

Among the full sample, Chosen Family DYK scores were negatively correlated with level of depressive symptoms (*r* = −0.19, *p* = 0.025), and positively correlated with the Cognitive Reappraisal subscale of the ERQ (*r* = 0.19, *p* = 0.024). Correlations between the Chosen Family DYK and Expressive Suppression subscale of the ERQ were not observed. Moreover, Cognitive Reappraisal scores were negatively correlated with level of depressive symptoms (*r* = −0.23, *p* = 0.006) Among SMW, Chosen Family DYK scores were positively correlated with Outness (*r* = 0.27, *p* = 0.008).

#### Testing Direct and Indirect Effects

We initially examined whether Cognitive Reappraisals played a mediating role in the relationship between Chosen Family DYK scores and level of depressive symptoms. Given that the number of free parameters in the model equaled the number of known values (the model had zero degrees of freedom), the model was just-identified. There was a significant indirect effect, such that higher Chosen Family DYK scores (knowing more about one’s chosen family) were associated with lower depressive symptom severity through higher Cognitive Reappraisal scores [*B* = −0.05, SE = 0.04, 95% CI (−0.13, −0.01)]. Next, we examined whether this association would be found among heterosexual women compared to SMW. The model demonstrated good fit based on the chi-square test of model fit, [χ^2^ (2, 147) = 5.67, *p* = 0.059] and *SRMR* = 0.06, and poor fit based on RMSEA = 0.11 90% CI (0.00–0.23) and CFI = 0.78. Considering the small sample size, RMSEA and CFI values are interpreted with caution and a larger sample size is likely needed to obtain more precise results (Kline, 2011; Kenny and Judd, 2014). Results of the moderated mediation analysis demonstrated that the influence of Cognitive Reappraisals on the association between Chosen Family DYK scores and level of depressive symptoms was negative for heterosexual women [*B* = −0.06, SE = 0.04, 95%CI (−0.16, −0.02)] but not SMW [*B* = −0.02, SE = 0.05 95%CI (−0.15, 0.03)], suggesting that heterosexual women compared to SMW benefited more from knowing about their chosen family, as this was associated with higher Cognitive Reappraisal scores and lower depressive symptoms.

**FIGURE 2:**
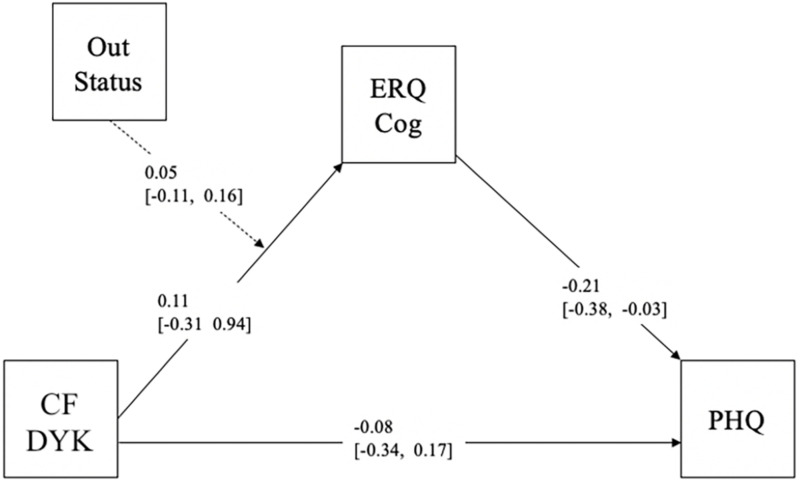
Overall model SMW. Out Status, Outness; CF DYK, Chosen Family Do You Know; ERQ Cog, Emotion Regulation Questionnaire-Cognitive Reappraisals; PHQ, Patient Health Questionnaire-9.

To further explore factors that may be affecting SMW’s mental health in the overall model, we examined outness as a moderator of the initial mediation model among SMW only (*n* = 98; see Figure 2). Findings revealed that the mediating role of Cognitive Reappraisals on the relationship between Chosen Family DYK scores and level of depressive symptoms was stronger for SMW who are out and open about their sexual identity with friends and family [*B* = −0.10, SE = 0.08, 95%CI (−0.30, −0.01)], but this effect was only trending for SMW who are moderately out and open [*B* = −0.08, *SE* = 0.06, 95%CI (−0.20, −0.00)] and the effect was not seen for SMW who are not as out and open [*B* = −0.05, SE = 0.06, 95%CI (−0.20, 0.00)].

## Discussion

This study was the first to examine whether knowledge of one’s family of origin history as well as one’s chosen family history are associated with level of depressive symptoms, a metric that influences wellbeing. These findings showed that only heterosexual women who knew more about their family of origin history showed lower levels of depression symptoms, but importantly, it was observed that knowledge of one’s chosen family history and level of depression were significantly correlated among both heterosexual women and SMW. Additionally, these data are the first to identify potential mediators that underlie this association with chosen family history. Among both heterosexual women and SMW, the employment of cognitive reappraisals as an emotion regulation strategy mediated the relationship between chosen family knowledge and level of depressive symptoms; however, this association was further mediated among SMW who reported higher levels of outness across various contexts. Taken together, these outcomes expand the potential sources of history that might be associated with positive mental health outcomes and important sociocultural and psychological factors that may contribute to these associations.

Of note, the results replicated the original findings between the DYK and metrics influencing wellbeing as reported by Fivush et al. (2008), but not among the full sample. Heterosexual women, even though they did not know more about their families of origin, showed this association with lower levels of depression symptoms. These findings suggest that there may be additional underlying factors that will help to illuminate why such relations are psychologically beneficial in some populations and not in others.

Interestingly, although there may be more stigma and conflict in families in which a member is SMW, there were no significant between-groups differences in terms of knowledge on the DYK. In contrast to our expectations, feminine-presenting SMW knew more about their families of origin than heterosexual women on a marginal basis. Although the small sample size may have prohibited statistically significant findings, this sample of feminine-presenting SMW may have strong relationships with their families of origin, either since childhood or as they have aged, in accordance with some literature that has found a tendency for parents to move toward accepting and supporting their SM children over time (Beals and Peplau, 2006; McConnell et al., 2016). Therefore, the work that may be involved moving toward acceptance, may involve memory practices and sharing that could contribute to these higher scores on the DYK, but this needs to be examined in further research. Since most participants resided in an especially progressive area of the country, it is also possible that experiences with familial rejection may not have been as common among this sample.

Although only heterosexual women replicated previous findings from Duke et al. (2008), all three groups of women who knew more about their chosen family reported lower depressive symptoms regardless of their sexual orientation. While the term *chosen family* has been traditionally associated with SM communities, socially constructed families (also referred to as voluntary kin) may represent an important aspect of human experience irrespective of sexual identity, as evidenced by reports of such relational ties dating back to the first century (Braithwaite et al., 2010). Further support is provided by evidence of the association between having quality extra-familial relationships and living longer (Chopik, 2017). To our knowledge, this is the first study to extend previous findings regarding knowledge of family history and metrics that influence wellbeing to a relationship outside of the family of origin. While connections to our family of origin are likely critical for healthy identity development and wellbeing in adolescence, our findings suggest that friendship plays an important role in sustaining wellbeing in adulthood for both SM and heterosexual populations, potentially for different reasons.

Substantial empirical evidence has demonstrated the importance of quality social support on wellbeing in general (Helliwell et al., 2009; Chopik, 2017), with links to higher life satisfaction, higher positive and lower negative affect, and lower psychological distress (Finch et al., 1999; Siedlecki et al., 2014). Social support is believed to impact the perception of one’s ability to cope with challenges, as supportive connections afford emotional resources that can aid in buffering stress (Cohen, 2004). Given that SMW and other marginalized populations face additional stressors relative to the general population, supportive connections may function in a variety of ways, including processing experiences of distal stressors, which may decrease the adverse effects of proximal stressors, such as internalized homophobia. Most importantly, relational, cognitive, and emotional processes involved in the ordinary conversations and shared activities occurring in supportive relationships may serve to regulate affect, thus enhancing mental health (Lakey and Orehek, 2011). Chosen families drive the formation of dense and diverse social support networks, which ultimately provide a defense against the negative effects of stigma-related stress (Moore and Stambolis-Ruhstorfer, 2013; McConnell et al., 2016).

In addition, our results identified emotion regulation as a mediator of the association between chosen family knowledge and level of depressive symptoms for heterosexual women; however, the extent of outness moderated the strength of this relationship for SMW. These findings are consistent with the extant literature covering the importance of emotion regulation processes in mental health, independent of sexual orientation (Aldao et al., 2010; Kring and Sloan, 2010). With regard to SMW, this finding serves as an important extension of the psychological mediation framework advanced by Hatzenbuehler (2009) in demonstrating the role of protective factors, like emotion regulation, on the better mental health experienced by SMW who are more open about their sexual identities. While further research is needed to clarify the mechanisms underlying this relationship, we postulate that SMW who are more out are more comfortable with their sexual identities owing to the quality of the supportive relationships in their lives, which translates into more emotional resources for processing distal stigma events, such as discrimination. SMW who are less out may not feel as comfortable or safe in their environments, which may leave them vulnerable to proximal stressors, such as internalized homophobia.

The findings of the present study emphasize the importance of social support networks, and the relational, cognitive, and emotional processes integral to them, on adult mental health, both for heterosexual and SMW, as the quality of friendships has been found to predict health and wellbeing in comparison to family of origin relationships, especially among older adults (Giles et al., 2005; Christakis and Fowler, 2007; Chopik, 2017). For SMW in particular, having access to a chosen family can mitigate stigma-related stress and plays a role in the development and/or quality of emotion regulation strategies, which have been connected to mental health. Future research should seek to clarify the nature of this relationship.

There are several methodological limitations that warrant attention. First, the present study is cross-sectional; therefore, we cannot make claims of causality cannot be made. Additionally, responses were self-reported and future work would benefit from interviews and the use of tasks to assess emotion regulation processes. Additionally, there may be a number of factors that contribute to knowledge of family history that were not assessed in this study, such as a history family trauma, family cohesion, communication dynamics, and attachment styles. The inclusion of such variables in future work will play an important role in better understanding the relations between family knowledge and wellbeing in families in which stigma and other barriers to family communication may be present. Considering that a majority of the sample was solicited *via* the Internet, participants may have been more likely to be educated and of a higher socioeconomic status. Lastly, the generalizability of our results are limited due to self-selection bias and the location of a majority of participants in New York City.

The underlying processes cognitive, emotional, and relational processes connected to sharing autobiographical memories within families of origin have been connected to wellbeing in adolescents (Duke et al., 2008). Of course, for many reasons, stories from one’s family of origin may not be available. However, these findings indicate that the notion of family can be extended with regard to these patterns. In the absence of, or in addition to, stories from family members of origin, stories that friends, romantic partners, and other members of one’s social support networks may offer may also be linked with lower levels of depressive symptoms, which factors into wellbeing. Given the relations between outness and emotion regulation, future clinical work would benefit from examining whether mental health interventions that combine chosen family knowledge along with emotion regulation strategies may help to reduce levels of depression.

## Data Availability Statement

The raw data supporting the conclusions of this article will be made available by the authors, without undue reservation.

## Ethics Statement

The studies involving human participants were reviewed and approved by Human Research Protection Program, The New School. The patients/participants provided their written informed consent to participate in this study.

## Author Contributions

JG and AB collaborated on the research design, interpretation of the results, and manuscript writing. JG conducted the research and was responsible for recruiting and managing interactions with participants. MP conducted the statistical analyses. WH provided guidance at several key junctures, including the conceptualization of the project and review of the final manuscript. All authors contributed to the article and approved the submitted version.

## Conflict of Interest

The authors declare that the research was conducted in the absence of any commercial or financial relationships that could be construed as a potential conflict of interest.

## Footnotes

1 The term *sexual minority* refers to individuals who identify as non-heterosexual in self-description (e.g., lesbian, bisexual, gay), sexual behavior, and sexual attraction.
